# Reproducibility of deep inspiration breath hold for prone left-sided whole breast irradiation

**DOI:** 10.1186/s13014-014-0313-4

**Published:** 2015-01-09

**Authors:** Thomas Mulliez, Liv Veldeman, Tom Vercauteren, Werner De Gersem, Bruno Speleers, Annick Van Greveling, Dieter Berwouts, Vincent Remouchamps, Rudy Van den Broecke, Wilfried De Neve

**Affiliations:** Department of Radiotherapy, Ghent University Hospital, De Pintelaan 185, 9000 Ghent, Belgium; Department of Radiotherapy, Clinique et Maternité Sainte-Elisabeth, Place Louise Godin 16, 5000 Namur, Belgium; Department of Gynaecology, Ghent University Hospital, De Pintelaan 185, 9000 Ghent, Belgium

**Keywords:** Breast, Radiotherapy, Prone position, Supine position, Deep inspiration breath hold, Reproducibility

## Abstract

**Background:**

Investigating reproducibility and instability of deep inspiration breath hold (DIBH) in the prone position to reduce heart dose for left-sided whole breast irradiation.

**Methods:**

Thirty patients were included and underwent 2 prone DIBH CT-scans during simulation. Overlap indices were calculated for the ipsilateral breast, heart and lungs to evaluate the anatomical reproducibility of the DIBH maneuver. The breathing motion of 21 patients treated with prone DIBH were registered using magnetic probes. These breathing curves were investigated to gain data on intra-fraction reproducibility and instability of the different DIBH cycles during treatment.

**Results:**

Overlap index was 0.98 for the ipsilateral breast and 0.96 for heart and both lungs between the 2 prone DIBH-scans. The magnetic sensors reported population amplitudes of 2.8 ± 1.3 mm for shallow breathing and 11.7 ± 4.7 mm for DIBH, an intra-fraction standard deviation of 1.0 ± 0.4 mm for DIBH, an intra-breath hold instability of 1.0 ± 0.6 mm and a treatment time of 300 ± 69 s.

**Conclusion:**

Prone DIBH can be accurately clinically implemented with acceptable reproducibility and instability.

## Background

Whole breast irradiation (WBI) after surgery in early-stage breast cancer patients has been related to secondary cancer induction and cardiac toxicity [1-3]. These complications may potentially reduce the shown benefits of WBI on overall survival [4]. Therefore, recent research in the field of breast radiotherapy has focused on techniques lowering the dose to the organs at risk (OARs) while maintaining an adequate dose to the ipsilateral breast. In supine position, the breast enwraps the heart and ipsilateral lung and is flanked by the contralateral breast permitting only limited beam access without traversing these OARs. Due to this proximity, dose reductions to one OAR without compromising dose to other OARs are only possible to a certain extent. Deep inspiration breath hold (DIBH) has been described in supine position to significantly lower heart dose metrics by increasing the heart-breast distance for patients receiving left-sided WBI [5-9]. An alternative to supine setup is prone position, which exploits anatomical changes due to gravitation and has been shown to significantly decrease lung dose in all patients and heart dose in the majority of patients compared to the standard supine position [10-14].

This trial is a part of a phase I-II study combining the advantages of DIBH and prone positioning for left-sided WBI. The mean heart dose was lowered from 2.2 Gy for prone normal or shallow breathing (SB) to 1.3 Gy for prone DIBH. Moreover the lung sparing ability of prone positioning was preserved (paper submitted). Dosimetric advantages of a novel treatment technique can only be extrapolated into a clinical benefit when accurate clinical execution can be guaranteed; this trial describes the reproducibility and instability of DIBH in the prone position for left-sided WBI.

## Methods

This study was designed as a prospective, mono-centric feasibility trial approved by the Ethics Committee of the Ghent university hospital. Thirty consecutive female left-sided breast cancer patients were included after informed written consent. These patients underwent breast-conserving surgery, were lymph node negative and eligible for adjuvant left-sided WBI.

All patients underwent CT-simulation in the prone-lateral position, which we further refer to as prone position for ease of reading. The first eight patients were used to gain experience with the DIBH maneuver in prone position and were treated in prone SB, the last 22 patients were accepted for prone DIBH WBI treatment. One of the 22 patients wasn’t able to perform prone treatment due to abdominal pain and was re-simulated and treated in supine position.

**Figure 1:**
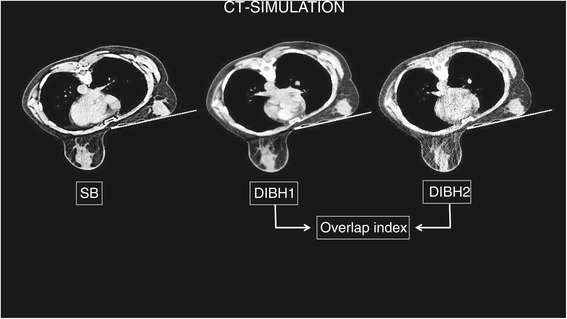
**During simulation, one prone shallow breathing (SB) followed by two prone deep inspiration breath hold (DIBH) CT-scans were taken without altering the scan range.** The second prone DIBH (DIBH2) CT-scan was acquired with adapted parameters to minimize radiation exposure to the patient. Overlap indices were calculated by rigid registration of DIBH1 and DIBH2.

Prone positioning was performed on a modified prone breast board (Orfit Industries, Wijnegem, Belgium) using a unilateral breast holder developed by Van de Velde (Schellebelle, Belgium) [13]. The patient’s breathing motion was registered using 2 Respisens magnetic sensors (Nomics, Angleur, Belgium) placed at the breast board and lateral thoracic wall [13,15,16]. The voluntary DIBH-maneuver consisting of two introductory non-deep breaths followed by a deep inspiration and a breath hold phase was reported previously by Remouchamps *et al.* [16]. The different DIBH cycles during simulation and treatment were instructed using verbal audio coaching. During simulation, one prone SB and two prone DIBH CT-scans were acquired as shown in Figure 1. Neither patient positioning nor scan range were altered, therefore assuring that the DICOM coordinate system, indicated by the frame of reference UID of the different scans, remained identical. The first DIBH scan (DIBH1) was used for treatment purposes. The second scan (DIBH2), with adapted CT-scan parameters to minimize radiation exposure to the patient, was used to verify the anatomical reproducibility of DIBH in the prone position. The images were transferred to a Pinnacle planning station (Philips Medical Systems, Andover, US) and delineation of the heart, both breasts and lungs was done on SB, DIBH1 and DIBH2 CT datasets as reported in previous publications [12,13,15,17]. Lung volumes were evaluated with the paired *t*-test. Rigid registration of the DIBH1 and DIBH2 CT-scans was done in order to evaluate the anatomical reproducibility of the DIBH maneuver. DIBH1 and DIBH2 were fused based on the DICOM coordinates and the overlap index was calculated for the ipsilateral breast, heart and both lungs. The overlap index was defined as the intersection of the volumes on DIBH1 (V_DIBH1_) and DIBH2 (V_DIBH2_) divided by the volume on DIBH1 (V_DIBH1_) [12].

$$ \mathrm{Overlap}\kern0.5em \mathrm{index}\kern0.5em ={\mathrm{V}}_{\mathrm{DIBH}1}\kern1em \cap \kern1em {\mathrm{V}}_{\mathrm{DIBH}2}/{\mathrm{V}}_{\mathrm{DIBH}1} $$

The higher the anatomical reproducibility, the higher the overlap index.

**Figure 2:**
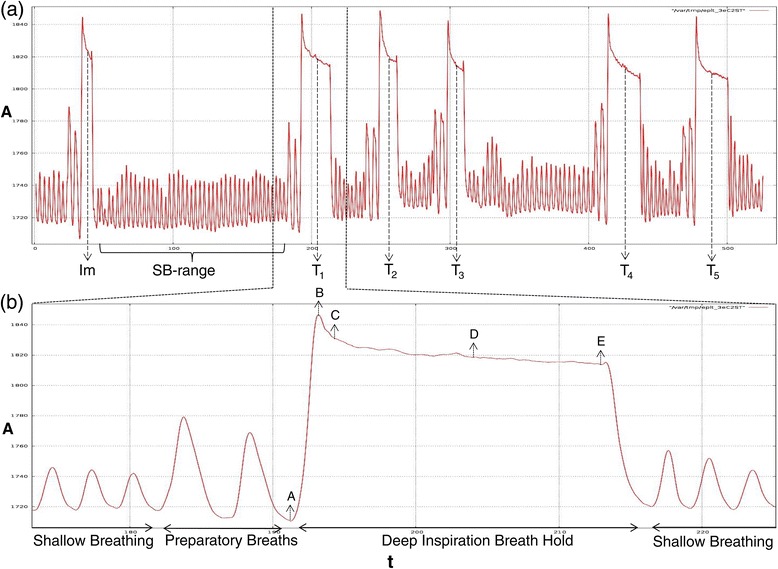
**Graphical output from the Respisens system of a typical sequence of prone deep inspiration breath holds (DIBH).** The horizontal axis defines time (t), the vertical axis amplitude (A). The upper part **(a)** demonstrates a typical breathing curve during treatment. kV-kV imaging (Im) was used to correct translational errors of DIBH (between Im-T_1_). Afterwards different DIBH-maneuvers (T_1_-T_5_) were performed for irradiation, note the gap between T_3_ and T_4_ to rotate the gantry. The SB-range was defined to calculate the SB-amplitude. The lower part **(b)** shows the details of a DIBH-maneuver with two preparatory breaths followed by deep inspiration and breath hold during approximately 15 seconds. A systematic peak is visible at the beginning (B) as well as a smaller peak at the end (after E). The following parameters, A till E, are assessed to gain data on prone DIBH amplitude, instability and time.

Twenty-one patients received WBI on an Elekta Synergy linear accelerator (Elekta, Crawley, West-Sussex, United Kingdom) to a prescription dose of 40.05 Gy in 15 fractions of 2.67 Gy. If required a sequential boost was given in 4 to 6 fractions according to the department’s guidelines. Figure 2a provides the typical breathing curve during one treatment session recorded with the Respisens probes. The breathing curves registered by the Respisens system was used to analyze the reproducibility and instability of the breath hold amplitude. In-house C++ software was used to analyze the Respisens data. The noise of the Respisens dataset was initially reduced using a symmetric 25-points Savitzky-Golay filter [18] and normalized to an average amplitude. A Cholesky decomposition was used to fit a second degree polynomial, which was subtracted from the amplitude to obtain a trend-corrected dataset, which was used to compute the maximum inspiration and expiration time per breathing cycle based on the method described by Veldeman *et al.* [15]. The amplitude of each inspiration and expiration time was determined on the non trend-corrected data.

Prone SB:

A shallow breathing range was selected to compute all minimum and maximum amplitudes. The SB amplitude was calculated as the difference between the average of all minima and maxima.

Prone DIBH:

Characteristically, as shown in Figure 2b, within one breath hold phase there are 2 peaks caused by the contraction and relaxation of the thoracic muscles; one prominent at the beginning (B-C) and one less pronounced at the end (after E). The time and amplitude of maximum expiration and inspiration (A and B, respectively) was computed based on the minima and maxima as explained above. The duration of the breath hold-range (B till E; without the end-peak) was measured for each non-imaging DIBH phase (Tn). Afterwards, the peak at the beginning (between B and C) of the breath hold was subtracted for each breath hold-range.

- The intra-breath hold instability was calculated as the difference between the upper (C) and lower (E) values inside a breath hold.
- The DIBH amplitude was defined as the difference between the average (D) of (C) and (E) and the end-expiration through (A) preceding the breath hold.

The average and standard deviation of the DIBH amplitudes and intra-breath hold instability were calculated for each treatment session to evaluate intra-fraction DIBH reproducibility and instability. The DIBH-time was registered from A till E for each DIBH-maneuver; the treatment time was recorded from A of the first (T_1_) till E of the last (Tn) DIBH phase.

## Results

Patient characteristics are presented in Table 1. All patients underwent CT-simulation including 2 prone DIBH CT-scans. The overlap index (mean ± standard deviation) between DIBH1 and DIBH2 was 0.98 ± 0.04 for the ipsilateral breast, 0.96 ± 0.06 for the heart, 0.96 ± 0.03 for lung left and 0.96 ± 0.04 for lung right. Total lung volumes were 2748 ± 452 cc, 4239 ± 810 cc and 4228 ± 802 cc for SB, DIBH1 and DIBH2, respectively. Lung volumes between both DIBH-scans did not differ (p = 0.7 by the paired *t*-test).

**Table 1:** **Patient characteristics**

**Patient**	**Age**	**BMI**	**Pack year**	**V**_**breast**_
1	52	24	0	775
2	66	29	0	1153
3	72	25	0	690
4	51	24	14	465
5	64	26	0	1937
6	47	21	20	1379
7	71	28	0	960
8	40	25	0	869
1	50	28	10	907
2	55	35	30	862
3	51	35	5	2372
4	66	31	0	2060
5	64	29	0	663
6	55	23	0	954
7	50	26	4	556
8	49	24	30	929
9	50	20	2	485
10	61	31	25	1101
11	49	27	30	1159
12	51	38	7	2372
13	54	31	36	1728
14	62	25	0	1001
15	64	29	20	737
16	44	31	14	1831
17	55	27	0	677
18	55	21	0	627
19	40	17	11	176
20	52	24	10	640
21	68	30	38	1284
#	57	39	14	2637

Thirty patients were included. All patients underwent deep inspiration breath hold (DIBH) cycles in the prone position during simulation. Twenty-two patients were accepted for prone DIBH treatment; one patient indicated with # wasn’t able to perform prone positioning during treatment. *Abbreviations: BMI = body mass index; Pack year = smoking history expressed as pack year; V*_*breast*_ *= breast volume.*

Twenty-one patients were treated with prone DIBH. The Respisens data are presented in Table 2. The population amplitude of the DIBH was 4 times larger than the SB, showing the ability of patients to perform a deep breath in prone position. The intra-fraction standard deviation of the DIBH amplitude was 1.0 ± 0.4 mm (range 0.5-1.9 mm). This illustrates the high reproducibility of breath hold amplitudes during one treatment fraction. The instability of the DIBH, i.e. the difference in breath hold amplitude between the beginning and the end of one breath hold (without the peaks), was <2 mm in 19/21 patients (range 0.1 – 2.9 mm) with a mean intra-fraction SD of <1 mm in all patients. The number of breath holds required to deliver the treatment ranged from 4 to 7, each lasting on average 16 ± 1 s. This resulted in a treatment time of 300 ± 69 s (range 231-445 s).

**Table 2:** **Respiration data recorded with the Respisens system during treatment**

**Patient**	**A**_**SB**_ **(mm)**	**A**_**DIBH**_ **(mm)**		**I**_**DIBH**_ **(mm)**		**N**	**t**_**DIBH**_	**t**_**T**_
	**Mean**	**Mean**	**Ia SD**	**Mean**	**Ia SD**		**(seconds)**	**(seconds)**
1	3.0	12.0	0.9	1.5	0.7	6	16 ± 2	385 ± 43
2	3.6	8.4	0.8	1.4	0.5	4	15 ± 1	235 ± 33
3	1.9	15.8	0.9	1.0	0.3	5	16 ± 1	292 ± 21
4	0.9	6.4	0.6	0.4	0.2	7	18 ± 2	445 ± 34
5	1.6	12.2	1.4	0.9	0.4	4	16 ± 1	239 ± 23
6	2.9	24.4	1.3	1.3	0.8	4	18 ± 2	291 ± 89
7	1.7	10.8	1.0	1.0	0.4	4	17 ± 2	242 ± 66
8	2.1	5.7	0.8	0.4	0.1	4	16 ± 2	238 ± 15
9	6.8	12.4	1.1	1.1	0.4	4	17 ± 1	231 ± 12
10	3.7	10.7	1.0	1.1	0.3	5	16 ± 1	308 ± 37
11	2.2	13.4	0.9	0.9	0.2	4	17 ± 0	234 ± 29
12	3.9	13.0	0.8	2.0	0.3	4	16 ± 1	247 ± 20
13	2.7	8.8	1.0	1.3	0.5	5	15 ± 1	288 ± 49
14	3.8	11.1	0.8	0.6	0.3	5	18 ± 2	407 ± 202
15	2.4	10.2	1.2	0.9	0.3	5	14 ± 0	345 ± 118
16	1.4	7.4	0.6	0.3	0.1	6	16 ± 2	305 ± 30
17	3.4	15.3	1.6	2.9	0.9	5	16 ± 1	314 ± 96
18	4.0	21.6	1.9	1.6	0.7	5	15 ± 1	438 ± 85
19	2.6	9.6	0.8	0.6	0.5	4	15 ± 1	231 ± 43
20	2.4	10.7	1.6	0.1	0.3	4	18 ± 1	256 ± 19
21	2.6	6.2	0.5	0.4	0.2	5	14 ± 1	338 ± 126
Population	2.8 ± 1.3	11.7 ± 4.7	1.0 ± 0.4	1.0 ± 0.6	0.4 ± 0.2	5 ± 1	16 ± 1	300 ± 69

Individual and population averages for mean and intra-fraction standard deviation (Ia SD) for shallow breathing (SB) amplitude (A_SB_), deep inspiration breath hold (DIBH) amplitude (A_DIBH_) and instability (I_DIBH_). The number of DIBHs required for treatment is indicated by N, mean and standard deviations are displayed for DIBH (t_DIBH_) and treatment time (t_T_).

## Discussion

RT is part of the standard treatment for early breast cancer after breast conserving surgery. Though it is also associated with severe side effects to heart, lungs and contralateral breast [1-3]. DIBH has been shown to be an effective technique to lower heart dose in supine position [5-9], while prone position is clearly the preferred technique for lung sparing [10-14]. By performing prone DIBH we were able to combine the advantages of both entities. This trial focuses on the reproducibility and instability of DIBH in the prone position during simulation and treatment.

Despite the heterogeneous patient group (Table 1); all 30 patients were able to perform prone breath hold maneuvers during simulation. One of the 22 patients addressed for prone DIBH treatment couldn’t tolerate prone position during treatment due to abdominal discomfort. Since prone DIBH treatment is expected to take more time than a standard prone treatment, complaints due to prone positioning including neck/shoulder/rib/abdominal pain [17,19-21] can be of more importance. Still 21 of the 22 patients were able to perform repetitive breath hold cycles (range 4–7) of on average 16 ± 1 seconds during treatment. A mean increase in total lung volume of approximately 50% was seen and breath hold amplitudes were on average 4 times higher than SB amplitudes, illustrating the feasibility of DIBH in the prone position.

Reproducibility and instability of supine DIBH appears to be in the order of a few millimeters as reported in different studies [22-25]. Our data suggest similar high reproducibility of the prone DIBH technique. Overlap indices of ≥0.96 for breast, heart and lungs indicate a high rate of intra-fractional anatomical reproducibility during simulation. There is a 4-fold increase in amplitude by performing DIBH compared to SB. The intra-fraction SD of the breath hold amplitude was less than 2 mm in all patients, illustrating the high reproducibility of the breath hold amplitudes during treatment. The instability of the amplitude during one breath hold was 1.0 ± 0.6 mm for the whole population, which is <10% of the DIBH amplitude. This instability is quite consistent indicated by the very low intra-fraction standard deviation of 0.4 mm. Data on inter-fraction setup accuracy is reported elsewhere (paper submitted).

Prone DIBH is more time consuming and burdensome due to the extra CT-scan during simulation, treatment plans in SB and DIBH, a longer setup verification procedure with cone beam CT scan in SB and kV-imaging in DIBH and longer treatment time. Further research is ongoing in order to validate these results and to improve these drawbacks.

## Conclusions

DIBH for prone left-sided WBI is achievable with adequate reproducibility and instability during simulation and treatment. Further research is needed to validate these results.

## Abbreviations

WBI: Whole breast irradiation
OARs: Organs-at-risk
DIBH: Deep inspiration breath hold
SB: Shallow or normal breathing
t: Time
A: Amplitude
Im: kV-kV imaging
T: DIBH cycle
BMI: Body mass index
Pack year: Smoking history expressed as pack year
V_breast_: Breast volume
Ia SD: Intra-fraction standard deviation
I: Instability
N: Number of DIBH cycles for treatment
t_DIBH_: DIBH time during treatment
t_T_: Treatment time
